# Cetuximab for esophageal cancer: an updated meta-analysis of randomized controlled trials

**DOI:** 10.1186/s12885-018-5040-z

**Published:** 2018-11-26

**Authors:** Ze-Hao Huang, Xiao-Wen Ma, Jing Zhang, Xiao Li, Na-Lin Lai, Sheng-Xiao Zhang

**Affiliations:** 1 Department of Head & Neck Surgery, Cancer Hospital, Peking Union Medical College, Chinese Academy of Medical Science, Beijing, 100021 China; 2 Department of Endocrinology, Xi’an Hong Hui Hospital, Xi’an, 710054 Shanxi Province China; 3 Department of Nursing, Shanxi Children’s Hospital, Taiyuan, Shanxi Province China; 4 Department of Rheumatology, The Second Hospital of Shanxi Medical University, 382 Wuyi Road, Taiyuan, 030001 Shanxi Province People’s Republic of China

**Keywords:** Cetuximab, Esophageal cancer, Epidermal growth factor receptor, Meta-analysis

## Abstract

**Background:**

Increasing evidence indicates that cetuximab (CET) combined with chemoradiotherapy may be effective for patients with esophageal cancer. However, the recent results are still contradictory and no consensus has yet been reached on this issue. To evaluate the clinical effects and safety of CET, we conducted an updated meta-analysis by retrieving published data up to June 2018.

**Methods:**

A comprehensive literature search was performed in several electronic databases, including PubMed, Embase, the Cochrane Library, CNKI database and Chinese Biomedicine Database using subject terms and free terms. Pooled odds ratios (ORs) with 95% confidence intervals (CIs) were calculated to determine the efficiency and safety of CET.

**Results:**

This meta-analysis included 10 randomized controlled trials (RCTs). Five RCTs reported localized esophageal cancer and other five RCTs reported metastatic esophageal cancer. For these patients with localized esophageal cancer, CET could not significantly improve the response rate, overall survival and progression-free survival (PFS, 1–5 years). But CET treatment might increase the incidences of diarrhea (OR = 2.07; CI = 1.01–4.25) and rash (OR = 16.91; CI = 3.20–89.42). For other patients with metastatic esophageal cancer, the addition of CET significantly increased the response rate (OR = 3.34; CI = 1.90–5.88), disease control rate (OR = 2.92; CI = 1.49–5.71) and 2-year overall survival (OR = 2.78; CI = 1.20–6.46) compared with the control group. However, CET could not improve the 1-year overall survival and might make patients with metastatic esophageal cancer more susceptible to rash (OR = 5.50; CI = 2.14–14.14). No significant differences in other adverse effects were found between the two groups.

**Conclusions:**

Our findings suggested that adding CET to multimodal therapy significantly improved response rate and disease control rate for patients with metastatic esophageal cancer rather than patients with localized esophageal cancer. CET might be a safe therapeutic choice, but CET failed to significantly improve the overall survival and PFS for patients with localized or metastatic esophageal cancer.

## Background

Esophageal cancer, mainly includes esophageal adenocarcinoma and esophageal squamous cell carcinoma (ESCC), is a kind of malignant tumor threatening human health seriously. It has become the 8th most common cancer type and the 6th leading cause of cancer mortality [1] with over 400, 000 deaths annually worldwide [2]. Despite increasing advances in the outcomes of many other solid tumors, the therapy of patients with esophageal cancer is still a big challenge for researchers and clinicians.

In the 1990s, the results of RTOG 85-01 established the standard care for the nonoperative treatment in localized esophageal cancer using concurrent chemoradiotherapy [3, 4]. Recent years, radiotherapy has been improved to reduce the injury of normal tissues and then yields a benefit for esophageal cancer patients [5, 6]. However, the standard therapy has not developed greatly due to the slow-moving chemotherapy in the last decades.

Increasing studies have found various biologic markers in esophageal cancer, including epidermal growth factor receptor (EGFR), vascular endothelial growth factor (VEGF) and HER-2 [7]. Among them, EGFR (also called HER-1) is one member of the HER family of tyrosine kinase receptors, which are encoded by the erbB oncogene. EGFR is overexpressed in approximately one third [8] to half [9] of esophageal adenocarcinomas and 71% of ESCC [10] and is associated with poor prognosis [11].

Cetuximab (CET), a monoclonal antibody of EGFR, could improve outcomes when given in combination with chemotherapy/radiotherapy in several tumors, including advanced colorectal adenocarcinomas [12], squamous-cell head and neck cancer [13] as well as esophageal cancer [14]. A previous meta-analysis published 3 years ago showed no effects of cetuximab combined with standard approaches for esophageal cancer by pooling results from three randomized controlled trials (RCTs) and two studies [15]. Thereafter, another several high-quality randomized controlled trials (RCTs) were published in this field and some discrepancies appeared. For example, Lu et al. argued that CET could improve clinical therapeutic effects,prolong survival time, and reduce therapeutic side effects in the treatment of intermediate and advanced esophageal cancer [16]. Therefore, it is necessary to conduct an updated meta-analysis by retrieving more recent publications to clarify the pooled effects of CET for esophageal cancer. In the present comprehensive review and meta-analysis, we summarized published data from all eligible RCTs to demonstrate the response, therapeutic effects and safety of CET in combination with conventional approaches.

## Methods

### Search strategy

We identified studies on the use of cetuximab in the treatment of esophageal cancer, published up to May 31, 2018. Electronic databases comprising PubMed, Embase, the Cochrane Library, CNKI database, and Chinese Biomedicine Database were comprehensively searched. Both subject terms and free terms were used as the search strategy to retrieve RCTs. The following keywords were used: “esophageal cancer”, “esophageal carcinoma”, “carcinoma of the esophagus”, “cetuximab”, “erbitux”, “C225”. Reference lists of obtained articles were also evaluated by hand. Conference abstracts meeting the inclusion criteria were also included and we tried to contact the authors to obtain original data. No publication date or language restrictions were adopted.

### Inclusion criteria

In the present meta-analysis, we used the following inclusion criteria: (1) only esophagus cancer trials have been included in the analysis, including esophageal squamous cell carcinoma (ESCS), adenocarcinoma or undifferentiated carcinoma of the esophagus, and adenocarcinoma of the thoracic esophagus. (2) Both metastatic and localized esophageal cancer were accepted. (3) Comparing the efficacy and safety of CET with CET-free treatment. (4) Reporting at least one of the following outcomes: overall survival, progression-free survival, response rate, disease control rate and side effects. (5) RCTs. It should be noted that all trials with +/− CET were included and then they were analyzed separately regarding the disease status (metastatic or non-metastatic).

### Exclusion criteria

Our exclusion criteria included (1) patients less than 20 subjects, (2) reviews and qualitative studies, (3) animal study and cell experiment and (4) duplicated reports.

### Data extraction

Two investigators, ZH Huang and XW Ma, independently reviewed all of the trails and extracted data. The primary data included (1) the name of first author and the publication year of each study, (2) date of patient enrolled, (3) country, (4) trail design, (5) disease, (6) arms, (7) sample size, age and gender of included patients, (8) follow-up duration and (9) main outcome measures, including overall survival, progression-free survival, response rate, disease control rate and side effects. Any discrepancy was discussed with another qualified reviewer (J Zhang) until reaching a consensus.

### Quality assessment

Evaluation of the bias risk of 10 eligible RCTs was conducted by two authors (ZH Huang and XW Ma) using the standard scoring criteria supported by the Cochrane Back Review Group. There are 5 domains (selection bias, performance bias, attrition bias, reporting bias, and detection bias) in this assessment form. Each domain contains one to three items, thus there are 12 items in total. Each item can be rated as “Yes”, “Unclear” or “No”, and the former scores 1 point, while the others score 0 points. A study scored a total of 8 or more points can be rated as a high-quality study. A low-quality study should score fewer than 6 points. Other studies are regarded as moderate-quality studies.

### Statistical analysis

We assessed the efficacy and safety of CET in the treatment of esophageal cancer based on the data from randomized controlled trials. Meta-analysis of variables was performed when the outcome was reported by two or more studies. The results, including overall survival, progression-free survival (PFS), response rate, disease control rate and incidence of adverse events, were treated as dichotomous variables, and they were reported as pooled odds ratios (ORs) with 95% confidence intervals (CIs) for each study. All these parameters were summarized separately based on the disease status. A fixed-effects model or random-effects model with Mantel-Haenszel weighting was used for estimating the pooled outcomes, depending on the heterogeneity between the included studies. Heterogeneity derived from included studies was tested using a Chi-squared based I^2^ statistics before the original data were synthesized [17]. If there was significant heterogeneity among studies, a random-effects model was used; otherwise, a fixed-effects model was used to summarize the pool data. When I^2^ ≥ 50% or *p* ≤ 0.1, the studies were considered to have significant heterogeneity [18]. Review Manager software for Windows version 5.3 (Cochrane Collaboration, Oxford, United Kingdom) was used for all calculations and statistical analyses. *p* ≤ 0.05 was considered as statistical significant. Publication bias and sensitivity analyses were not conducted because of the limited number of included studies [19, 20].

## Results

### Study selection

**Fig. 1:**
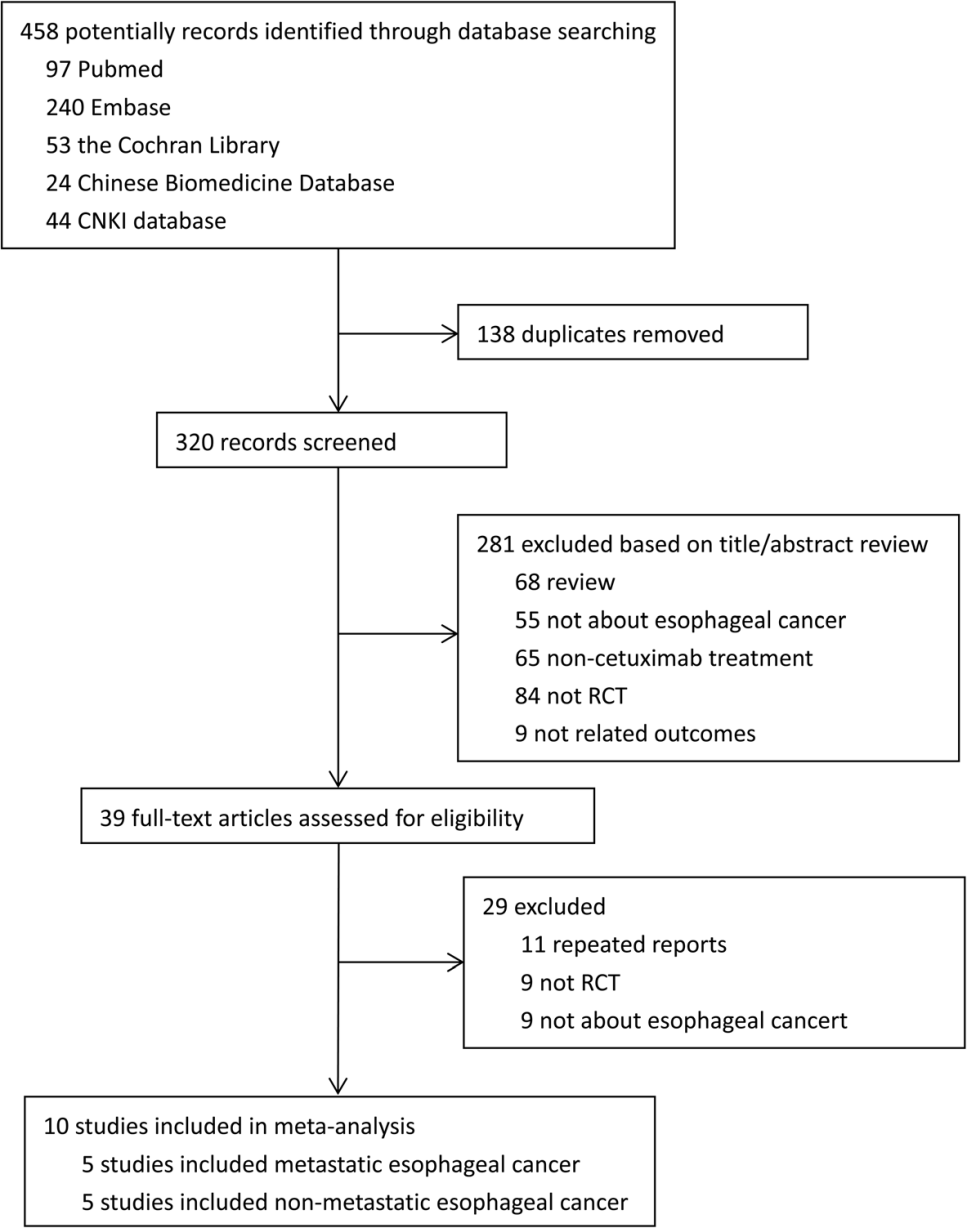
PRISMA flow chart of study selection in Meta-analysis

We initially obtained 458 studies from Embase, PubMed, the Cochrane Library, CNKI database, and Chinese Biomedicine Database. After removing the duplicated studies in EndNote X7 software, 320 potentially relevant studies were identified as candidates for a review based on title/abstract. The remaining 39 studies were further evaluated by the full text. We excluded 29 studies due to the following reasons: 11 were repeated reports with the same data, nine were not RCTs and nine were not about esophageal cancer. Thus, 10 RCTs [14, 16, 21–28] with 1346 patients with esophageal cancer that met the inclusion criteria were eventually included in the present meta-analysis. The Literature selection process was performed under the guideline of PRISMA (Fig. 1).

### Study characteristics

All 10 included trials were well performed, prospective randomized controlled trials published from 2009 to 2018. All studies consisted of two arms, i.e., CET and CET-free treatment in combination with chemotherapy or radiotherapy or chemoradiotherapy. Most eligible studies were conducted in Europe [14, 21, 23, 27] and China [16, 22, 24–26], and one study was from the United States of America [28]. The average age of patients with esophageal cancer was between 50 and 70 years old. Male participants were far more than female participants. Follow-up duration among these RCTs varied from 1 to 72 months. Moreover, five trials included localized esophageal cancer [21, 23, 24, 27, 28] and other five trials included metastatic esophageal cancer [14, 16, 22, 25, 26]. For these studies about localized esophageal cancer, four trials reported overall survival [21, 24, 27, 28], two trials reported progression-free survival [27, 28], two trials reported response rate [23, 28], one trial reported disease control rate [23] and two trials reported the side effects appeared during treatment [27, 28]. For these studies about metastatic esophageal cancer, three trials reported overall survival [14, 16, 25], one trial reported progression-free survival [14], five trials reported response rate [14, 16, 22, 25, 26], three trials reported disease control rate [14, 16, 25], and two trials reported the side effects appeared during treatment [14, 16]. Most studies about metastatic esophageal cancer used CET combined with chemotherapy, while only Feng et al. [25] adopted chemoradiotherapy. The detailed characteristics of the included studies are presented in Table 1.

**Table 1:** Characteristics of 11 included studies

Subtype classification of esophageal cancer	Study (year)	Design	Disease	Arms	Country	Years enrolled	Population (N, age)	Sex: Male/Famale	Follow-up duration (months)	Primary end-point	Main outcome measures
Non-metastatic esophageal cancer	Rades et al. (2014) [23]	RCT; open-label, randomized multicenter phase II study	Unresectable locally advanced esophageal cancer	Radiochemotherapy with 5FU, cisplatin, 59.4 Gy/6.5 weeks plus/minus cetuximab	Germany	NM	*N* = 20, NM	NM	24	Response rates	Response, PFS and survival, disease control rate
	Zhang et al. (2014)	RCT; unicentre, randomised, parallel, two-arm trial	Localized esophageal cancer	Routine chemotherapy, routine chemotherapy plus cetuximab	China	2008–2009	*N* = 80, 46–79 years old	52/28	36	Overall survival	Overall survival, recurrence rate, transfer rate
	Crosby et al. (2017) [21]	RCT; multicentre, randomised, open-label, parallel, two-arm, phase 2/3 trial	Localized esophageal squamous cell cancer and adenocarcinomas	CRT only, CRT plus cetuximab	UK	2008–2012	*N* = 258, 67 (35.7–84.1) years old	145/113	60	Overall survival	Compliance, toxicities, PFS and survival, causes of death
	Ruhstaller et al. (2017)	RCT; multicentre, randomized, open-label phase III trial	Locally advanced but resectable ESCC and thoracic esophagus adenocarcinomas	Neo-adjuvant chemotherapy followed by chemoradiation (45 Gy, docetaxel 20 mg/m^2^ and cisplatin 25 mg/m^2^, weekly for 5 weeks) and surgery with and without cetuximab	Switzerland, Germany, Austria, France	2010–2013	*N* = 300, 61 (53–68) years old	263/37	72	PFS	Compliance, surgery and pathological remission rate, efficacy, safety
	Suntharalingam et al. (2017) [28]	RCT; multicentre, randomized, phase III trial	locally advanced ESCC or adenocarcinoma of the esophagus or gastroesophageal junction	RT (daily radiation of 50.4 Gy/1.8 Gy fractions) + Chemo, RT + Chemo + cetuximab	USA	2008–2013	*N* = 328, 64 (57–71) years old	276/52	36	Overall survival	Tolerance and toxic effects, survival, response, local failure
Metastatic esophageal cancer	Lorenzen et al. (2009) [14]	RCT; multicenter, open-label, noncomparative randomized phase II study	Nonresectable, advanced ESCC, including metastatic disease	CF, CET-CF	Germany	2004–2006	*N* = 62, 61 (40–76) years old	52/10	24	The confirmed objective response rate	Response, safety and tolerability, PFS and survival
	Chen et al. (2014) [22]	RCT; unicentre, randomised, parallel, two-arm trial	Advanced esophageal cancer, incuding metastatic disease	CRT only, CRT plus cetuximab	China	2011–2012	*N* = 40, 57.3 ± 5.3 years old	26/14	12	PFS	Response, safety, PFS and survival
	Feng et al. (2017) [25]	RCT; unicentre, randomised, parallel, two-arm trial	Thoracic esophageal carcinoma with lymph node metastasis	Radiotherapy (2.5–3.5 Gy/time, 4–5 times/week, 65–70 Gy in total) plus chemotherapy, radiotherapy plus cinobufotalin and cetuximab	China	2011–2013	*N* = 78, 59.35 ± 6.08 years old	43/35	36	Overall survival	Response, overall survival, quality of life, serum indicators
	Yang et al. (2017) [26]	RCT; unicentre, randomised, two-arm trial	Advanced esophageal cancer, incuding metastatic disease	Chemotherapy, chemotherapy plus cetuximab	China	2016–2017	*N* = 100, 37–77 years old	61/39	1	Response rates	Response, toxic effects
	Lu et al. (2017) [16]	RCT; unicentre, randomised, parallel, two-arm trial	Advanced esophageal cancer, incuding metastatic disease	Chemotherapy with cisplatin and 5FU, Chemotherapy with cisplatin and 5FU plus cetuximab	China	2013–2015	*N* = 80, 45–80 years old	51/29	12	Overall survival	CEA, SCC, response, overall survival

Abbreviations: *RCT* randomized controlled trial, *CF* cisplatin and fluorouracil, *CET–CF* cetuximab, cisplatin and fluorouracil, *PFS* progression-free survival, *CRT* chemoradiotherapy, *ESCC* esophageal squamous cell carcinoma, *5FU* 5-fluorouracil, *RT* radiation therapy, *CEA* carcino embryonic antigen, *SCC* squamous cell carcinoma antigen, *NM* not mentioned

### Study quality

All included studies were RCTs, which could be considered relatively high-quality. According to the standard scoring criteria, for these trials about non-metastatic esophageal cancer, one study [21] scored 8 points and could be regarded as high-quality. While two studies [23, 24] scored 5 points and should be regarded as low-quality. The remaining two studies [27, 28] scored 7 points and should be regarded as moderate-quality. For the trials about metastatic esophageal cancer, one study [22] scored 4 points and should be regarded as low-quality. The remaining four studies [14, 16, 25, 26] scored 6–7 points and should be regarded as moderate-quality. Most studies lost points because they failed to state the method of random sequence generation, or did not adopt blinding. The summary of the risk of bias was presented in Table 2.

**Table 2:** Quality assessment of the included studies

Bias	Selection bias			Performance bias			Attrition bias			Reporting bias (no selective reporting)	Detection bias		Study quality (score)
Study	Adequate random sequence generation	Similar baseline condition	Adequate allocation concealment	Adequate participant blinding	Adequate provider blinding	Similar or no co-interventions	Acceptable compliance	Acceptable drop-out rate	intention-to-treat analysis		Adequate outcome assessor blinding	Similar timing of outcome assessment	
Studies included non-metastatic esophageal cancer													
Rades et al. (2014) [23]	Unclear	Unclear	Unclear	Unclear	Unclear	Yes	Yes	Unclear	Yes	Yes	Unclear	Yes	Moderate (5)
Zhang et al. (2014)	Unclear	Unclear	Unclear	Unclear	Unclear	Yes	Unclear	Yes	Yes	Yes	Unclear	Yes	Moderate (5)
Crosby et al. (2017) [21]	Yes	Yes	Yes	No	Unclear	Yes	No	Yes	Yes	Yes	Unclear	Yes	high (8)
Ruhstaller et al. (2017)	Unclear	Yes	Unclear	Unclear	Unclear	Yes	Yes	Yes	Yes	Yes	Unclear	Yes	Moderate (7)
Suntharalingam et al. (2017) [28]	Unclear	Yes	Unclear	Unclear	Unclear	Yes	Yes	Yes	Yes	Yes	Unclear	Yes	Moderate (7)
Studies included metastatic esophageal cancer													
Lorenzen et al. (2009) [14]	Unclear	Yes	Unclear	Unclear	Unclear	Yes	Yes	Yes	Yes	Yes	Unclear	Yes	Moderate (7)
Chen et al. (2014) [22]	Unclear	Yes	Unclear	Unclear	Unclear	Yes	Unclear	Unclear	Yes	Yes	Unclear	No	Low (4)
Feng et al. (2017) [25]	Yes	Yes	Unclear	Unclear	Unclear	Yes	Unclear	Yes	Yes	Yes	Unclear	Yes	Moderate (7)
Yang et al. (2017) [26]	Unclear	Yes	Unclear	Unclear	Unclear	Yes	Unclear	Yes	Yes	Yes	Unclear	Yes	Moderate (6)
Lu et al. (2017) [16]	Yes	Yes	Unclear	Unclear	Unclear	Yes	Unclear	Yes	Yes	Yes	Unclear	Yes	Moderate (7)

### Overall survival

**Fig. 2:**
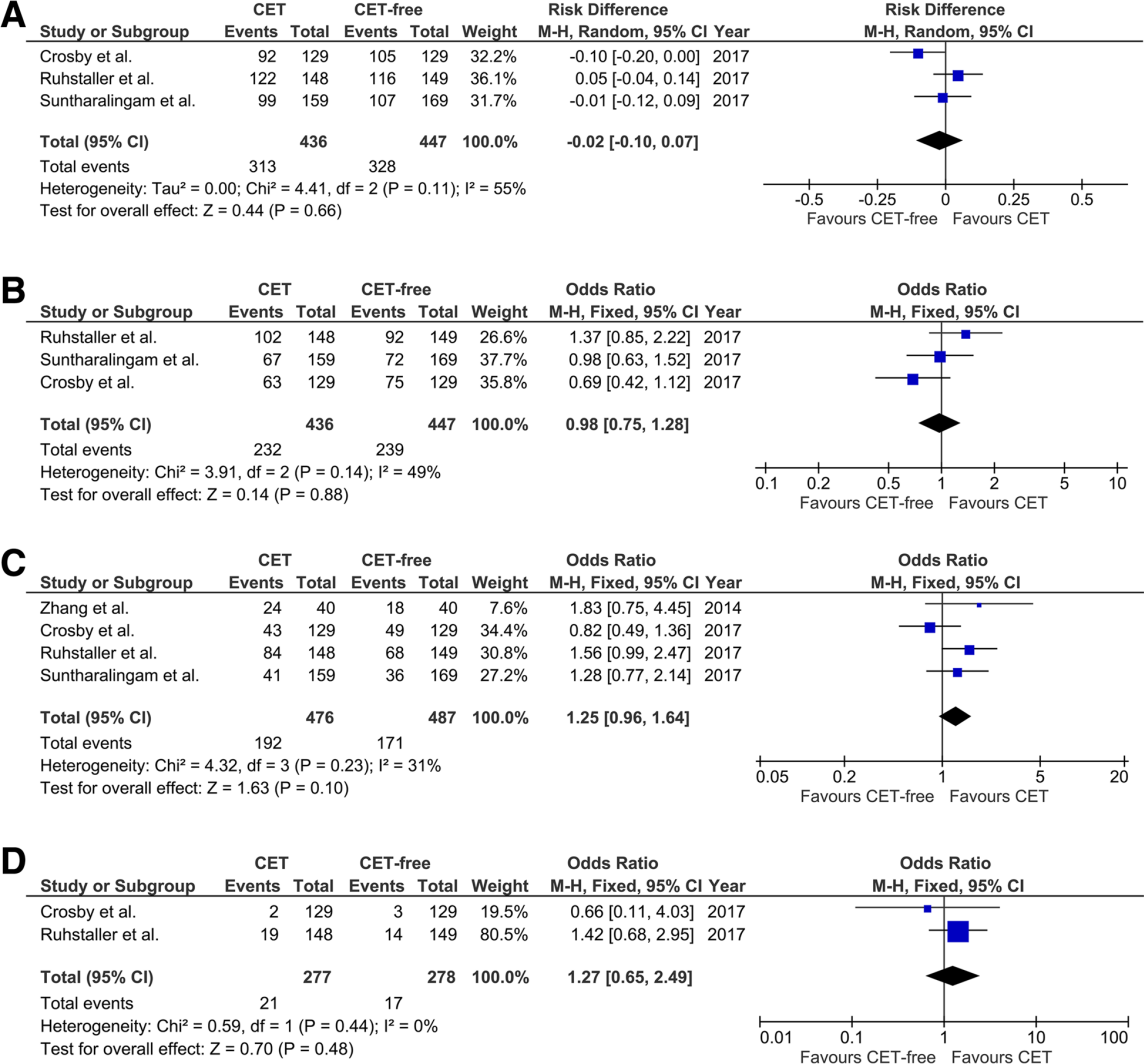
Forest plots of pooled overall survival in patients with localized esophageal cancer. **a** 1-year overall survival. **b** 2-year overall survival. **c** 3-year overall survival. **d** 5-year overall survival. CET, cetuximab

The effects of CET on overall survival were evaluated separately for patients with advanced (metastatic) esophageal cancer and patients with the locally advanced disease. For these patients with localized esophageal cancer, no significant effects of CET were found in 1-year (OR, − 0.02; 95% CI, − 0.10 to 0.07; *p* = 0.66; Fig. 2a), 2-year (OR, 0.98; 95% CI, 0.75 to 1.28; *p* = 0.88; Fig. 2b), 3-year (OR, 1.25; 95% CI, 0.96 to 1.64; *p* = 0.10; Fig. 2c), and 5-year overall survival (OR, 1.27; 95% CI, 0.65 to 2.49; *p* = 0.48; Fig. 2d). It should be noted that only two trials provided data on 5-year overall survival, while four trials provided data on 3-year overall survival. The different quantity of included studies may result in different power.

**Fig. 3:**
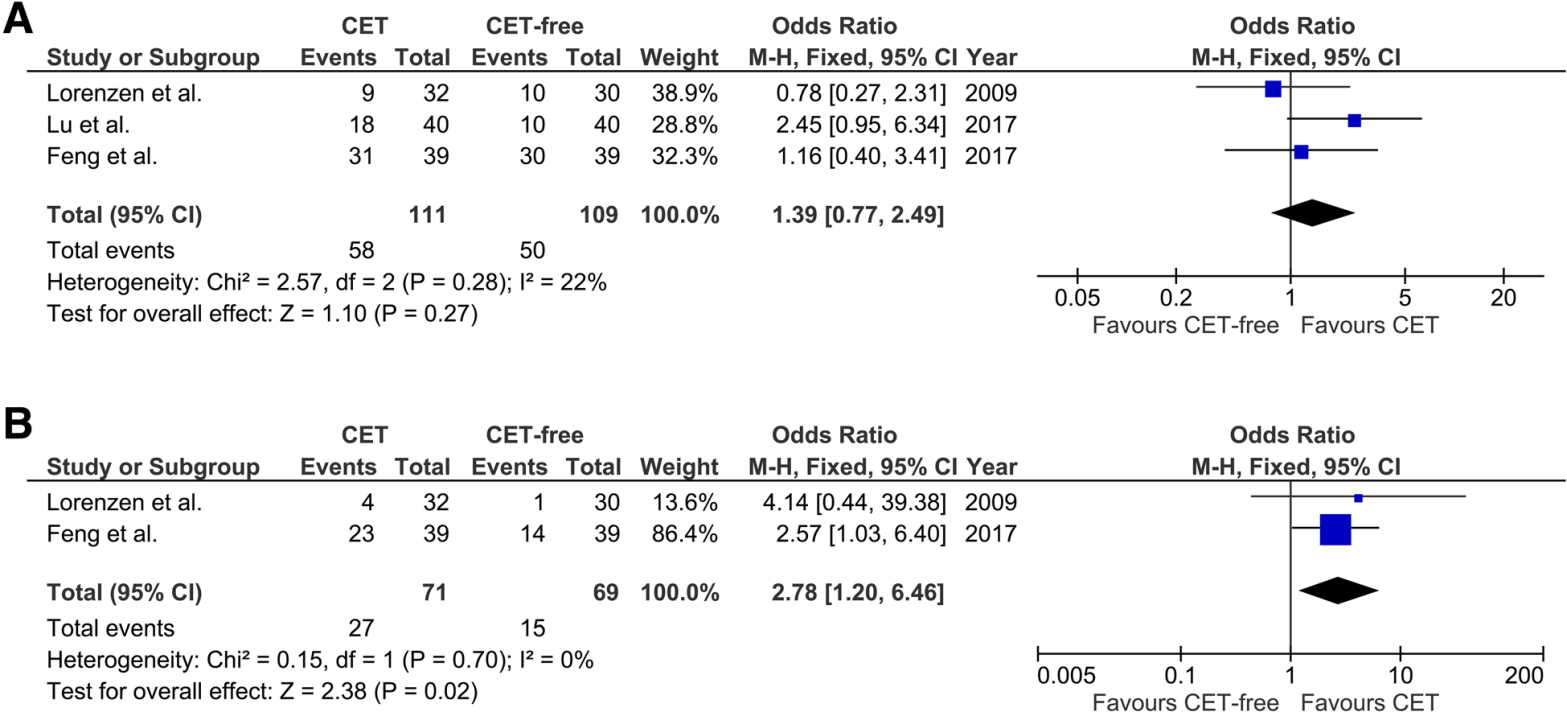
Forest plots of pooled overall survival in patients with metastatic esophageal cancer. **a** 1-year PFS. **b** 2-year PFS. CET, cetuximab

For other patients with metastatic esophageal cancer, CET also could not improve 1-year overall survival (OR, 1.39; 95% CI, 0.77 to 2.49; *p* = 0.27; Fig. 3a). However, CET could improve 2-year overall survival (OR, 2.78; 95% CI, 1.20 to 6.46; *p* = 0.02; Fig. 3b). The results of the meta-analysis of 3-year and 5-year overall survival were not calculated because there are less than two studies involved metastatic esophageal cancer.

### Progression-free survival (PFS)

**Fig. 4:**
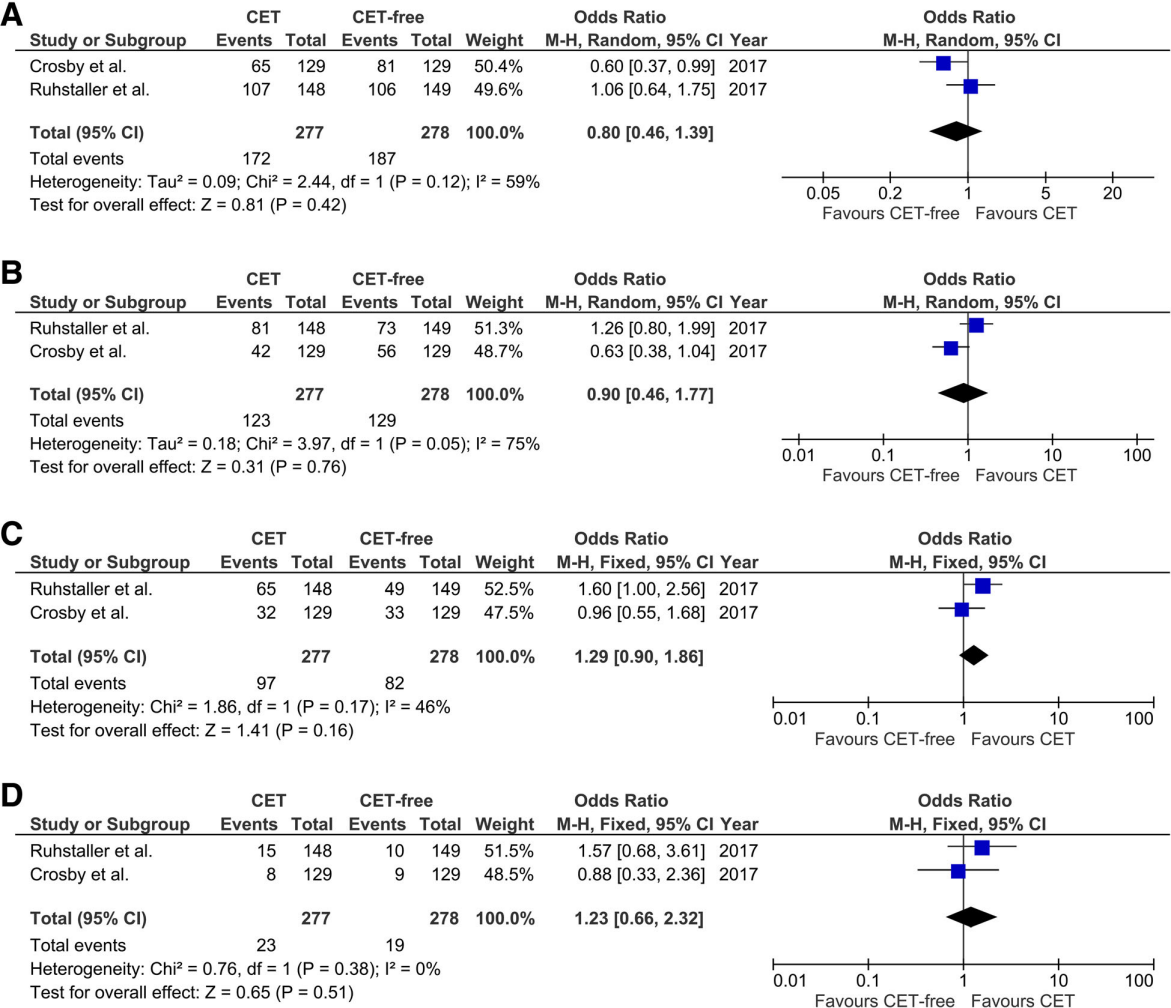
Forest plots of pooled PFS in patients with localized esophageal cancer. **a** 1-year PFS. **b** 2-year PFS. **c** 3-year PFS. **d** 5-year PFS. PFS, progression-free survival; CET, cetuximab

**Fig. 5:**
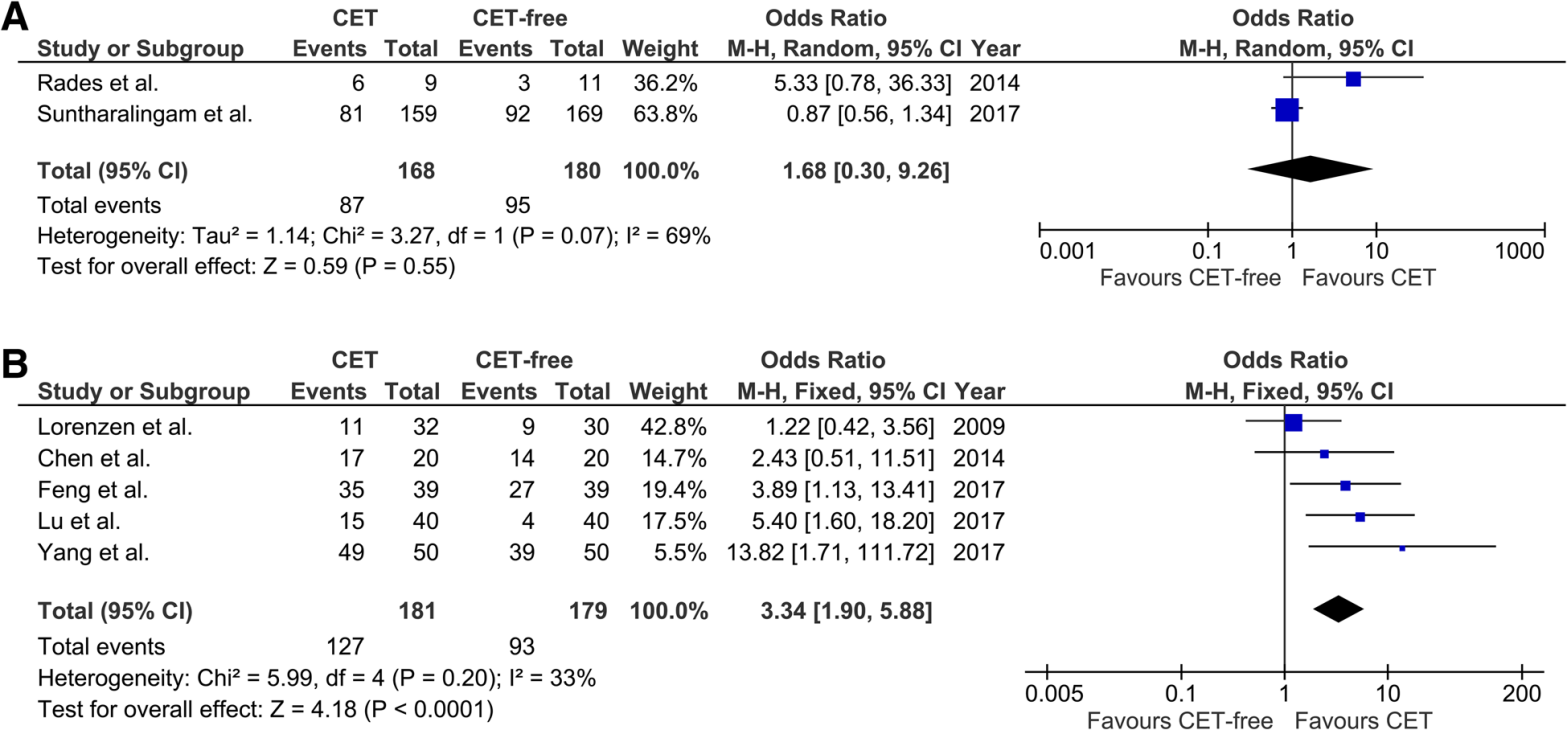
Forest plots of pooled response rate in patients with esophageal cancer. **a** Forest plots of pooled response rate in patients with localized esophageal cancer. **b** Forest plots of pooled response rate in patients with metastatic esophageal cancer. CET, cetuximab

Two studies reported the PFS of patients with localized esophageal cancer [21, 27], while only one study reported the PFS of patients with metastatic esophageal cancer [14]. Therefore, we only analyzed the 1-year, 2-year, 3-year or 5-year PFS of patients with localized esophageal cancer after the treatment with and without CET. As shown in Fig. 4, there is a significant heterogeneity in 1-year PFS (I^2^ = 59%, *p* = 0.12; Fig. 4a) and 2-year PFS (I^2^ = 75%, *p* = 0.05; Fig. 4b), but not 3-year PFS (I^2^ = 46%, *p* = 0.17; Fig. 4c) and 5-year PFS (I^2^ = 0%, *p* = 0.38; Fig. 4d). No significant differences were found between two groups in all the comparisons of PFS (all *p* > 0.05, Fig. 4).

### Response rate

Two studies with 348 patients with localized esophageal cancer reported the response rate [23, 28]. There was a significant heterogeneity among the studies in the comparison of response rate (I^2^ = 69%, *p* = 0.07; Fig. 5a). The pooled results showed that there was no significant difference between CET-administrated patients and CET-free patients (OR, 1.68; 95% CI, 0.30 to 9.26; *p* = 0.55; Fig. 5a).

Five studies with 360 patients with metastatic esophageal cancer reported the response rate [14, 16, 22, 25, 26]. The response rate in the original articles ranged from 34.38% [14] to 89.74% [25] in CET group and 10.00% [16] to 70.00% [22] in CET-free group. No significant heterogeneity was found among these studies in the comparisons of response rate (I^2^ = 33%, *p* = 0.20, Fig. 5b). The pooled results showed that the response rate was significantly higher in CET-administrated patients compared with the CET-free group (OR, 3.34; 95% CI, 1.90 to 5.88; *p* < 0.0001).

### Disease control rate

**Fig. 6:**
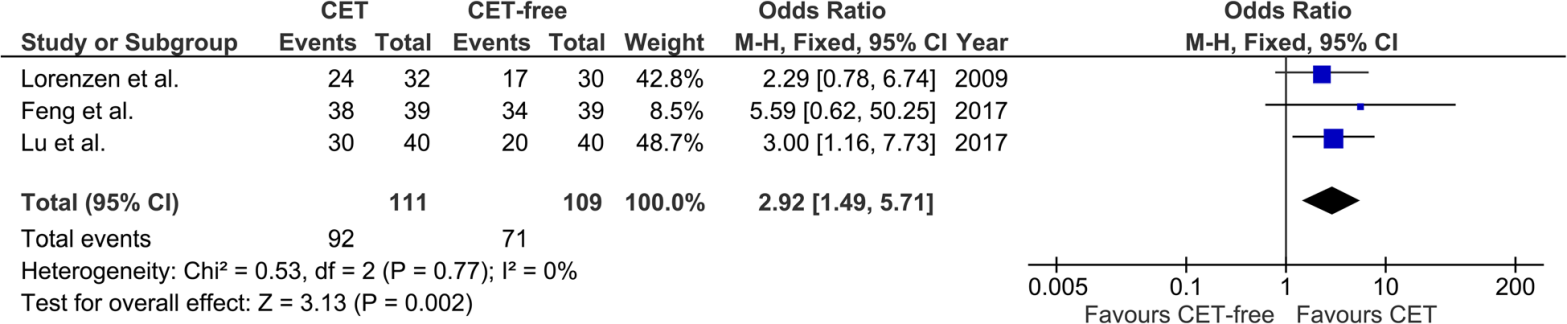
Forest plots of pooled disease control rate in patients with metastatic esophageal cancer. CET, cetuximab

Three studies reported the disease control rate of patients with metastatic esophageal cancer [14, 16, 25], while only one study reported the disease control rate of patients with localized esophageal cancer [23]. Thus, we only analyzed the disease control rate from the 3 studies with 220 patients with metastatic esophageal cancer. There was no significant heterogeneity among these studies (I^2^ = 0%, *p* = 0.77; Fig. 6). Pooling studies that compared disease control rate got an OR of 2.92 (95% CI, 1.49 to 5.71), which demonstrated notable benefit effects of CET for patients with metastatic esophageal cancer (*p* = 0.002; Fig. 6).

### Adverse effects (AEs)

Meta-analysis of AEs was performed when the outcome was reported by at least two studies. Various AEs were discussed during treatment in seven included RCTs [14, 21, 22, 25–28]. As shown in Table 3, we summarized the data of AEs based on the disease status. For these patients with localized esophageal cancer, the pooled results showed that CET-treated patients suffered more from diarrhea (OR, 2.07; 95% CI, 1.01 to 4.25; *p* = 0.05) and rash (OR, 16.91; 95% CI, 3.20 to 89.42; *p* = 0.0009) compared with CET-free-treated participants. No significant differences were found in the incidence of the remaining AEs (all *p* > 0.05), including nausea, emesis, fatigue, neuropathy, cardiac disorders, metabolic/laboratory, dyspnoea, infection, vascular disorders, acute kidney injury, and dysphagia.

**Table 3:** Pooled results of side effects in patients during treatment

Studies	Variables	No. of studies	No. of subjects	Meta-analysis		Model	Test of heterogeneity	
				OR (95% CI)	*p* value		I^2^	p value
Studies included non-metastatic esophageal cancer	Diarrhea	2	577	2.07 [1.01, 4.25]	0.05^*^	F	0	0.35
	Nausea	2	577	0.84 [0.48, 1.46]	0.53	F	16%	0.28
	Emesis	2	577	0.90 [0.48, 1.70]	0.75	F	4%	0.31
	Fatigue	2	577	1.22 [0.78, 1.92]	0.38	F	0	0.47
	Neuropathy	2	577	0.69 [0.30, 1.56]	0.37	F	0	0.45
	Rash	2	577	16.91 [3.20, 89.42]	0.0009^*^	F	0%	0.57
	Cardiac disorders	3	877	2.28 [0.78, 6.62]	0.13	F	0	0.47
	Metabolic/laboratory	2	577	1.78 [0.91, 3.51]	0.09	R	58%	0.12
	Dyspnoea	2	577	1.74 [0.62, 4.88]	0.29	F	28%	0.24
	Infection	2	577	1.48 [0.80, 2.74]	0.21	F	43%	0.19
	Vascular disorders	2	543	1.61 [0.82, 3.17]	0.17	F	38%	0.20
	Acute kidney injury	2	619	1.38 [0.31, 6.25]	0.67	F	0	0.84
	Dysphagia	2	619	0.88 [0.51, 1.53]	0.65	F	0	0.97
Studies included metastatic esophageal cancer	Rash	2	142	5.50 [2.14, 14.14]	0.0004^*^	R	92%	0.0004

Abbreviations: *OR* odds ratio, *F* fixed effects model, *R* random-effects model, *CI* confidence interval

*Statistically significant values (*p* ≤ 0.05), favors CET free

For other patients with metastatic esophageal cancer, only rash was reported in at least two studies. The meta-analysis of rash also showed that CET-treated patients were more susceptible to rash compared with CET-free-treated participants (OR, 5.50; 95% CI, 2.14 to 14.14; *p* = 0.0004).

## Discussion

Increasing evidence has indicated that CET may help treat various cancers, including non-small cell lung cancer (NSCLC) [21], colorectal adenocarcinomas [12] and squamous cell head and neck cancer [13], especially esophageal cancer. In this systematic review and meta-analysis, we retrieved published articles and summarized the evidence regarding the effects of CET in patients with esophageal cancer. EXPAND [29] and REAL3 [30] are two important phase-III trials which reported the effects of mAb against EGFR for patients with esophagogastric cancer. But they were excluded in the present meta-analysis because they also included gastric cancer. After a comprehensive search, 10 RCTs with 1346 patients with esophageal cancer were collected in our meta-analysis. Based on the outcomes reported in these included RCTs, we pooled the data of overall survival, PFS, response rate, disease control rate and various AEs according to the disease status. After pooling various evidence, this meta-analysis did not reveal that CET could significantly contribute to the increase of overall survival and PFS (1–5 years) in localized esophageal carcinoma. For these patients with metastatic esophageal cancer, no significant effect of CET was found in 1-year overall survival. However, 2-year overall survival in the CET group was significantly higher than the CET-free group in patients with metastatic esophageal cancer.

Moreover, in line with most studies, our meta-analysis demonstrated that CET could increase the response rate and disease control rate in patients with metastatic esophageal cancer. On the other hand, no significant effects of CET on response rate were found in localized esophageal carcinoma. Besides efficiency and response, the drug toxicity and safety also need great attention. Our meta-analysis results showed that the incidence of most AE complications during treatment was similar between CET-treated group and the control group. However, compared with control group, patients with localized or metastatic esophageal carcinoma were more likely to suffer from diarrhea and rash in the CET-treated group.

The outcome of our study is consistent with a previous meta-analysis [15] in this field, which has been published 3 years ago. Xu et al. compared the survival and safety between the CET and standard-approaches-based regimens [15]. This study only included five articles with only 401 patients. Generally, the limited power from a small sample may lead to false-negative results or cover false-positive results. Recent years, increasing studies have been performed to explore the effects of CET on patients with esophageal cancer, including phase III trials [27, 28]. The present updated meta-analysis summarized results from 10 studies with 1346 participants, which has a greater power. Besides, Xu et al. [15] included both RCTs (three) and case-control studies (two) in the previous meta-analysis, while only RCTs were qualified in the current study. The results from higher-quality studies are more convincing. Moreover, localized esophageal carcinoma and metastatic esophageal carcinoma were analyzed separately in the present meta-analysis, which helps to distinguish the different effects of CET in patients at different disease status.

Although CET has been shown to be safe when combined with chemotherapy and may increase the efficacy of standard chemotherapy [14], we did not find significant improvements in overall survival and PFS in esophageal carcinoma. A possible explanation is that some negative interactions may occur between CET and chemoradiotherapy or other standard approaches [31]. CET may reduce the efficacy in combination with chemoradiation for rectal cancer due to its anti-tumor and pro-inflammatory effects [32]. Besides, A similar interaction between CET and oxaliplatin has also been suggested in human colorectal cancer cells. Specifically, CET might limit the free-radical damage by platinum drugs [33]. Thus, the negative interaction may explain the non-significant outcomes. Future studies in this field may choose other non-platinum chemotherapy drugs to get rid of the negative interaction. On the other hand, it is also a good way to explore the potential effects of the analogs of CET for esophageal cancer. In addition, most trials in the non-metastatic group investigated regionally advanced, non-resectable disease [21, 28], while the SAKK trial included resectable disease [27]. The stage of the disease seems important, the SAKK trial with curatively-intended treatment was almost positive, compared to the more advanced disease in the non-metastatic group without surgery [27].

About 50–70% of patients with esophageal squamous cell carcinoma were accompanied by high expression levels of the epidermal growth factor receptor (EGFR) [8, 34]. EGFR has also been correlated with prognosis of esophageal cancer [35, 36]. Although CET is an immunoglobulin G1 mAb that specifically targets the EGFR, the addition of CET to concurrent chemoradiation did not improve survival. Most studies included in our meta-analysis did not focus the attention on EGFR expression. It is easy to understand that esophageal cancer patients without high EGFR expression may not benefit from EGFR inhibition. Actually, Zhang et al. [24] argued that CET could significantly increase the overall survival and reduce the recurrence rate and metastatic rate in patients with high EGFR expression, but did not significantly affect these outcomes in patients without high EGFR expression. Further research about CET should pay more attention to these esophageal cancer patients with over-expressed EGFR.

Despite this inaction in survival, the types and rates of AEs in our meta-analysis were consistent with those expected from the individual agent. No evidence showed that CET aggravated the known toxic effects of these standard approaches for patients with esophageal cancer. But the incidences of diarrhea and rash were higher in the CET-administrated group compared with the CET-free group. Moreover, Lorenzen et al. [14] stated that no deaths were reported to be related to CET. However, Crosby et al. [31] reported 3 treatment-related deaths in CET arm, while no treatment-related deaths were reported in the CET-free arm.

Overall, this study has several strengths. Firstly, the present meta-analysis was performed and reported according to the standard PRISMA guidelines. The standardized format is readable and comprehensible for other colleagues in this field. Secondly, no language restriction was set when we searched the published studies, and five Chinese articles [16, 22, 24–26] were identified and finally included in this study. Thus, our participants were from the European, Asia, and America. Thirdly, as stated before, our systematic review summarized data from 10 RCTs with 1346 participants. Most RCTs were of moderate quality, which guarantees the power of the test. Furthermore, we analyzed the heterogeneity before pooling ORs. Random-effects models were utilized when there is a significant heterogeneity. The conservative estimate contributes to a precise conclusion.

However, there are also several limitations in this meta-analysis. Firstly, three of the 10 trials reported the median with 95% CI or mean with standard deviation, but did not provide Kaplan-Meier curves for survival [22–24]. These missing data may lead to publication bias and finally affect the pooled results. Secondly, the control conditions are different in various original studies. Although chemoradiotherapy was commonly used as the standard approach for esophageal cancer, the treatment doses and durations were quite different. For example, the chemotherapy used by Ruhstaller et al. [27] consisted of cisplatin 75 mg/m^2^ and docetaxel 75 mg/m^2^ for 3 weeks, while Lorenzen et al. adopted chemotherapy with cisplatin 100 mg/m^2^ for 29 days followed by 5-fluorouracil 1000 mg/m^2^ for another 5 days [14]. Finally, the heterogeneity in some comparations is notable. Subgroup analyses is a good way to decrease heterogeneity, but subgroup analyses of different treatment (chemotherapy and chemoradiation) or cancer types (squamous cell carcinoma, adenocarcinoma, and undifferentiated carcinoma), CET dose and treatment duration were not performed because of the limited reports.

## Conclusions

In conclusion, the findings of the present updated meta-analysis suggested that adding CET to multimodal therapy significantly improved response rate and disease control rate for patients with metastatic esophageal cancer instead of patients with localized esophageal cancer. CET might be a safe therapeutic choice, but CET failed to significantly improve the overall survival and PFS for patients with localized or metastatic esophageal cancer. Further studies may concentrate on the efficacy of CET in esophageal cancer patients with high-expressed EGFR.
